# Distribution modeling of nanoparticles for brachytherapy of human eye tumor

**DOI:** 10.1186/s40658-020-00321-y

**Published:** 2020-08-20

**Authors:** S. Farhad Masoudi, Fahimeh S. Daryabari, Fatemeh S. Rasouli

**Affiliations:** Department of Physics, K.N. Toosi University of Technology, P.O. Box 15875-4416, Tehran, Iran

**Keywords:** Brachytherapy, COMS eye plaque, Gold nanoparticles (GNPs), Cell model, Dose enhancement factor (DEF), Monte Carlo simulation

## Abstract

**Background:**

Due to their unique properties, gold nanoparticles (GNPs) have been proposed to be used for a wide range of applications, especially for photon radiation therapy. In addition to experimental works, there are worthwhile simulation-based studies focused on the investigation of the effect of parameters governing the dose enhancement due to the presence of GNPs in tissue. In a recently published study, we found that the distribution of GNPs in a single cell plays an important role in nucleus dose enhancement.

**Methods:**

The present work investigates the sensitivity of dose enhancement of a macroscopic phantom to the modeling of GNPs at the cellular level by using the MCNPX Monte Carlo code. A human eye phantom containing the realistic structures and materials was simulated, with a typical tumor located in its corner filled with three different patterns of distribution of GNPs around the nuclei of the cells. The primary photons emit from a COMS eye plaque brachytherapy containing thirteen ^131^Cs seeds in the vicinity of the tumor.

**Results:**

The study was extended to estimate dose enhancement for various concentration, size, and density of the GNPs accumulated around the nuclei of the tumor. Moreover, the dose delivered to the healthy eye structures for different models has been investigated and discussed. The results show obvious differences between the dose enhancements in the tumor depending on the modeling of GNPs.

**Conclusion:**

The results emphasized that an appropriate small-scale model for the distribution of GNPs in the cell would be of high importance to estimate the degree of dose enhancement in a macroscopic phantom to provide a trustworthy prediction to move towards clinical application.

## Background

Increasing the efficiency of the brachytherapy [1] is possible to selectively increase the dose delivered to the target of interest during the radiation. Thanks to the promotion of nanotechnology in medicine, gold nanoparticles (GNPs) have become appropriate candidates to be used in cancer diagnosis and its treatment [2–7]. Due to the interaction between X-ray and high-Z constituent atoms of GNPs, the produced secondary electrons cause the increment in the energy deposited to the target. The effects of using GNPs as radiosensitizers on dose enhancement have extensively been studied in both experimental environments and Monte Carlo simulations. The commonly used term, dose enhancement factor (DEF) quantifies the magnitude of dose enhancement. DEF is defined as the ratio of the absorbed dose to the desired volume in the presence of GNPs to the absorbed dose in the same volume in the absence of nanoparticles.

There are excellent works, accomplished using both experimental [2, 8, 9] and simulation-based [10–12] approaches, dealing with the investigation of the effect of various parameters governing the DEF value. The simulation-based studies published so far have considered nanoparticles as gold-water (or gold-tissue) mixture or as the homogeneously distributed spheres in the medium [13–16]. However, the images reported in experimental documents show that the injected nanoparticles tend to accumulate around the cell nucleus [17–22]. Accordingly, the fundamental question is whether the exact simulation of the accumulation of nanoparticles in the medium plays a considerable role in the values of DEF, or gold-water mixture and the homogeneous distribution of GNPs in the medium lead to sufficient results.

There are pioneering studies in the literature which have examined the effect of the approach used for simulating nanoparticles on dose enhancement. For example, by simulating both gold-water mixture and homogeneously distributed GNPs, Zhang et al. [23] investigated the difference between the results corresponding to the mentioned models. They found about 36% dose enhancement for the gold-water mixture and about 28% dose enhancement for a homogenous distribution of gold nanospheres in the tumor. A similar study by Martinov and Thomson [24] showed that the homogeneous distribution of GNPs decreases the values of DEF by about 20% compared to the gold-tissue mixture. Moreover, several studies have inquired into the effect of utilizing different models for the distribution of GNPs at the cellular level. Sung et al. [25] investigated the effect of the cell geometry, and radiosensitization and the biological effectiveness of GNPs distributed in the extracellular media on the nucleus DEF. The study accomplished by Cai et al. [26] evaluates the effects of cell model, subcellular location, size, and the number of GNPs per cell, as well as the photon energy on the nuclear dose enhancement factor (NDEF). The results showed that the localization of GNPs in the nucleus and the increment of their number are two important factors for the growth of NDEF. In the study presented by Xie et al. [27], using a cell model with detailed DNA structure in the central nucleus, the physical and biological radiosensitization effect within the nucleus was set up and nanoparticles were distributed in different regions of the cell. They found that the enhancement in energy deposition increases in the case of GNPs with a diameter of 100 nm distributed on the nucleus surface. Considering the widely documented tendency of GNPs to localize around the nucleus, a recently published study has been devoted to investigate the importance of modeling GNPs distribution in a single cell on cellular dose enhancement and to examine the differences in the behavior of the parameters governing GNPs photon radiosensitization of cell nucleus arisen from using different models [28].

Although the mentioned studies try to evaluate the importance of using appropriate small-scale models of the distribution of GNPs in the cell on accurate estimation of cellular dose enhancement, however, the effect of distribution model of GNPs in the cell on macroscopic dose enhancement needs to be investigated in detail and to be well quantified. Inspired by the previous researches, the present work aims to investigate the sensitivity of the simulation results to the cellular modeling of nanoparticles in a typical macroscopic phantom. While the GNPs’ dose-enhanced radiotherapy of the eye has been the subject of several studies, the importance of the location of nanoparticles has not been investigated. To address this issue, a cell model containing nucleus and cytoplasm is designed, and three different patterns for distribution of GNPs are proposed: the gold-tissue mixture, homogeneously distributed GNPs in the medium, and the heterogeneous model with GNPs localized in the cytoplasm around the nucleus. These cells are considered to be located in a typical tumor of a simulated adult eye phantom including anatomical structures filled with realistic compositions. Several radioactive seeds of ^131^Cs that sit within the plaque with a specific geometry are also simulated in the vicinity of the tumor with the aim of brachytherapy. To show the gold concentration per gram of tissue, the notation of mg/g is introduced. Simulations and particle tracking in the medium have been carried out using MCNPX (Version 2.6.0) [29] Monte Carlo code.

## Methods

### Monte Carlo simulation

In the present work, we benefit from the Monte Carlo method, known as an appropriate way to simulate complex systems with many coupled degrees of freedom which involve the transport of particles through matter, to find the effect of the distribution of GNPs in the medium on the dose delivered to the tumor. Among the several codes commonly employed in nuclear physics problems, the MCNPX (Version 2.6.0) code has been chosen mainly due to its flexibility and possibility to use for solving different problems. Owing to that the Monte Carlo method is based on repeated random sampling and statistical analysis, the adequate number of histories, between 10^8^ and 10^9^, have been used to obtain the results with acceptable relative uncertainties. The uncertainties corresponding to the results have been calculated by applying the error propagation rules on the Monte Carlo relative errors. Variance reduction methods have not been used in the simulations, and the cut-off energies are the default values of the MCNPX code (1 keV for electrons). Detailed descriptions of the geometries, energies, and models simulated are presented in the following subsections.

### ^131^Cs seed (model CS-1)

Brachytherapy is a form of internal radiotherapy in which sealed radioactive sources are placed inside or near to the target volume to kill cancer cells and shrink the tumors. This method is extensively used for the treatment of uveal melanoma, one of the most frequent malignant intraocular tumors in adults. The procedure includes presurgical testing, determining the size of the plaque and the duration of time that the patient will need to wear the plaque by the radiation oncologist, stop taking or change in the dose of some of the medications, plaque implantation, treatment, and plaque removal. The American Brachytherapy Society (ABS) recommends a prescription dose of 85 Gy to the apex of the tumor [30]. It is proposed that a dose rate of 0.60 to 1.05 Gy/h delivers the total dose in 3 to 10 consecutive days [31]. To avoid the damage to the critical structures such as optic nerve, the dose ranging between 30 and 60 Gy has been suggested in the literature [32, 33].

While ^125^I and ^103^Pd seeds are commonly used in eye plaque brachytherapy implants, the advantages of ^131^Cs have made it as an appropriate candidate to be used for this purpose [34, 35]. The half-life of 9.7 days, and the average energy of 30.4 keV, which are respectively shorter than and higher than those of ^125^I and ^103^Pd seeds (see Table 1), can be listed as the advantages of this source. According to the literature [28, 37], the primary energy of about 30 keV leads to the highest value of DEF. In the present study, we have simulated the new ^131^Cs brachytherapy seed model CS-1 developed by IsoRay medical Inc [36]. A single CS-1 seed consists of cylindrical gold wire with a diameter of 0.25 mm and a length of 4.1 mm that is surrounded by a glass and ceramic material with a diameter of 0.65 mm has been coated with ^131^Cs. The seed is encapsulated inside a titanium tube with a diameter of 0.8 mm and a length of 4.5 mm [38]. According to the data published by National Nuclear Data Center (NNDC), the photons’ spectrum of ^131^Cs with their intensity is as follows: 4.11 keV (8.6%), 29.461 keV (21.1%), 29.782 keV (38.9%), 33.562 keV (3.63%), 33.624 keV (7.02%), 34.419 keV (2.13%) [39]. The activity of each seed has been considered to be 4 mCi.

**Table 1.** The data corresponding to the ^125^I, ^103^Pd, and ^131^Cs radioactive sources as commonly used seeds for eye plaque brachytherapy [36]

Data	^131^Cs	^103^Pd	^125^I
Half-Life (days)	9.7	17	60
Mean energy (keV)	30.4	20.8	28.5

### Models for distribution of GNPs

To investigate the effect of the distribution of GNPs on the nucleus dose enhancement, three various models are proposed:

i) Gold-tissue mixture. In this model, the material around the nucleus is considered as a homogeneous mixture of tissue and gold with a specific concentration. Owing to that the GNPs are injected in the tumor volume, the compositions of the tissue are considered to be those of eye melanoma [40].
ii) Homogeneously distributed GNPs in the medium. In this model, the spheres of GNP have homogeneously been distributed in the whole medium and in the cytoplasm. The remainder volume, including the nucleus and space between the GNPs, was defined as tumor tissue.
iii) The heterogeneous model. In this model, the GNPs are localized inside the cytoplasm and around the nucleus.

**Fig. 1.**
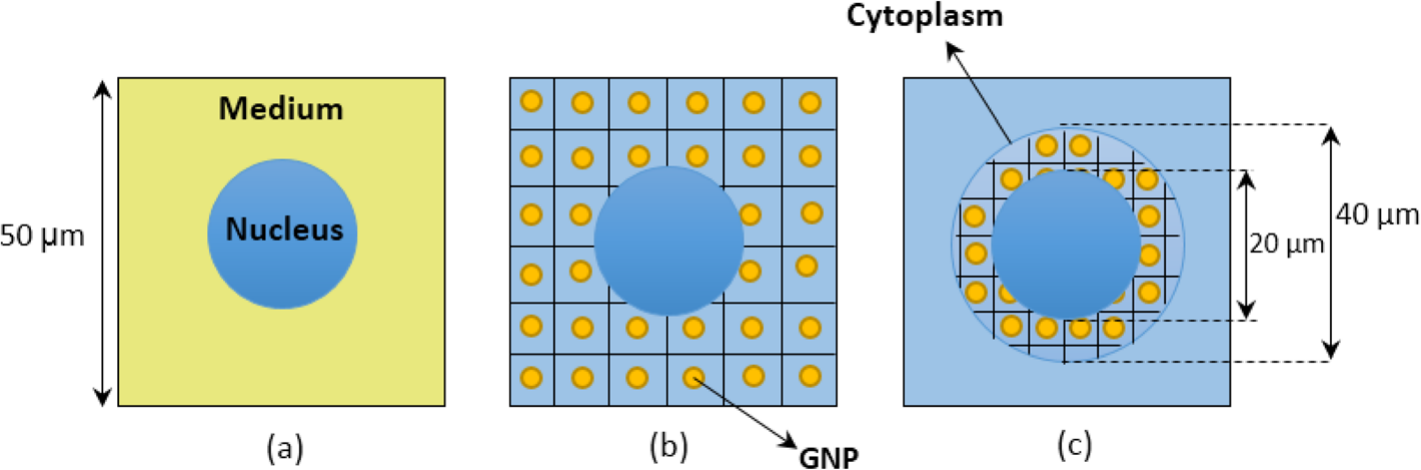
A schematic cross-sectional view of the models considered for distribution of GNPs in the cell. **a** Gold-tissue mixture. **b** Homogeneously distributed GNPs in the medium. **c** Heterogeneous model, with GNPs distributed inside the cytoplasm surrounding the nucleus. In the mixture model, the medium is filled with a homogeneous mixture of eye melanoma and gold. In all three models, the medium is a cubic box of 50 × 50 × 50 μm^3^, and the nucleus is filled with water

Schematic diagrams of a single cell, modeled with each of the foregoing patterns, have been shown in Fig. 1. According to the specified dimensions, which have been estimated based on the in vitro images reported by Rezaei Kanavi et al. [41], the tumor of our simulated phantom contains ~ 26 × 10^6^ cells. These cells have been simulated using the availability of simulating the repeated-structures by using the FILL, UNIVERSE, and LAT cards in MCNPX code. Owing that the damage to the DNA in the cell nucleus results in cell death, the physical doses due to the irradiation of photons are scored in the nucleus of each cell using *F8 tally. It is worth to mention that this work does not deal with the calculation of either the dose or damage delivered to the DNA and subcellular structures of a single nucleus. The values of the dose reported in this work are the mean dose delivered to the nuclei of the simulated macroscopic tumor.

### The eye phantom

**Fig. 2:**
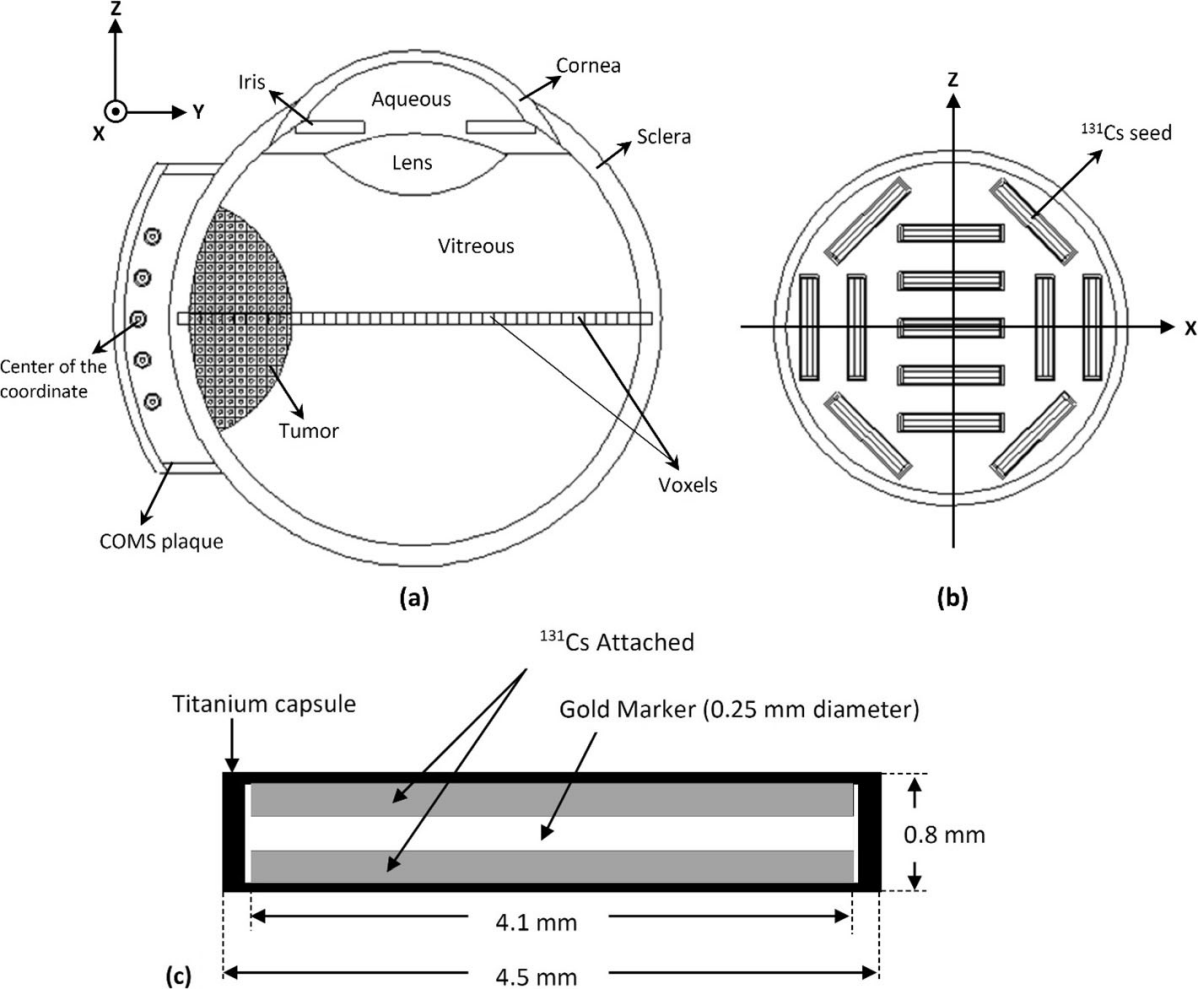
**a** Schematic cross-sectional view of the three-dimensional eye phantom including COMS plaque and tumor. The voxels along the *Y*-axis are designed for depth-dose calculations. **b** The simulated 14 mm (inner diameter) eye plaque containing thirteen ^131^Cs seeds, with various positions and orientations. **c** The geometry, materials, and dimensions of a single ^131^Cs seed

A geometrically and compositionally realistic three-dimensional model of the human eye developed by Lesperance et al. [40] is used as phantom. The dimensions, the shapes of ocular structures, and the elemental composition of the eye are also taken from the mentioned study. This phantom is representative of an adult eye and includes two concentric spheres as the outer and inner sclera, a hollow cylinder as iris, two spherical shells limited by sclera as the cornea, and a volume bounded by two spheres as the lens. In this model, a typical tumor, simulated as a sphere of 12 mm in diameter, is located in the corner of the phantom so that its apex lies 6 mm inward from the inner edge of the sclera. More details can be found in Ref. [42], and a schematic representation of the simulated phantom is sown in Fig. 2a. As the figure shows, the tumor volume is filled with the cells containing GNPs with the models explained in the previous subsection.

### COMS eye plaque brachytherapy

The radioactive seeds of ^131^Cs that sit within the plaque with a specific geometry are simulated in the vicinity of the tumor. This method, known as the collaborative ocular melanoma study (COMS) eye plaques, is common in treating eye tumors. The size of COMS eye plaques ranges between 10 and 22 mm, depending on the size of the ocular tumor. In the present study, a typical 14 mm inner diameter COMS eye plaque containing thirteen ^131^Cs seeds, with various positions and orientations, is used. The seeds are fixed by the silastic carrier with a density of 1.12 g cm^−3^ and are covered by gold alloy (77% gold, 14% silver, 8% copper, and 1% palladium) with the thickness of 0.5 mm and the density of 17.4 g cm^−3^ [43]. The simulated plaque is shown in Fig. 2b, and its location near the tumor in our human eye phantom are presented in Fig. 2a. The geometry and dimensions of a single ^131^Cs seed are also shown in Fig. 2c.

## Results and discussion

**Fig. 3:**
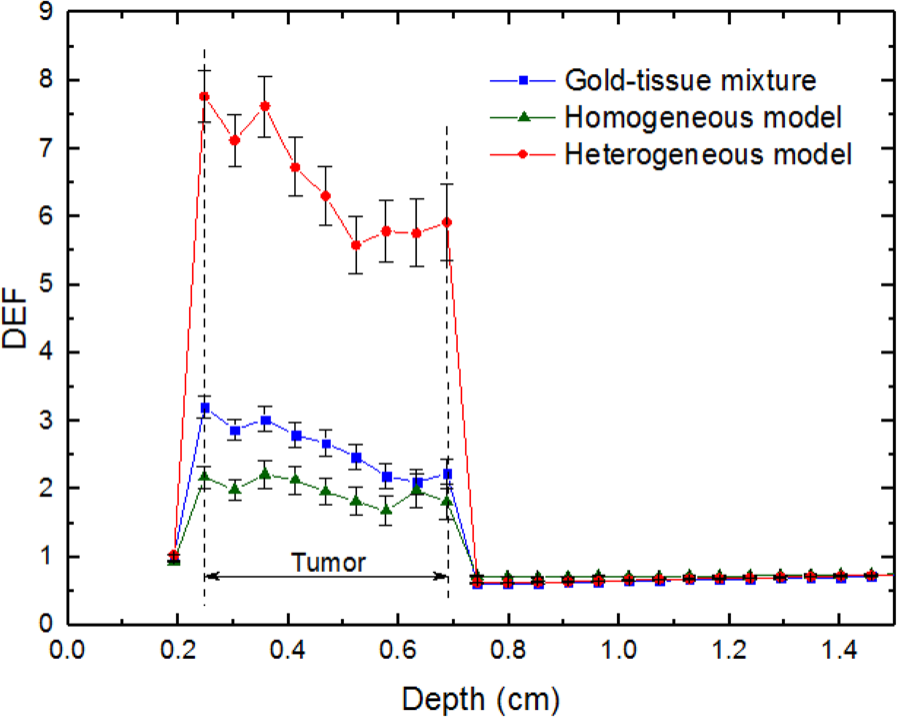
The values of DEF in the depth of human eye phantom (see Fig. 2a) containing a tumor filled with 100 nm GNPs with a concentration of 30 mg/g of different models. Error bars indicate the relative uncertainties

**Fig. 4:**
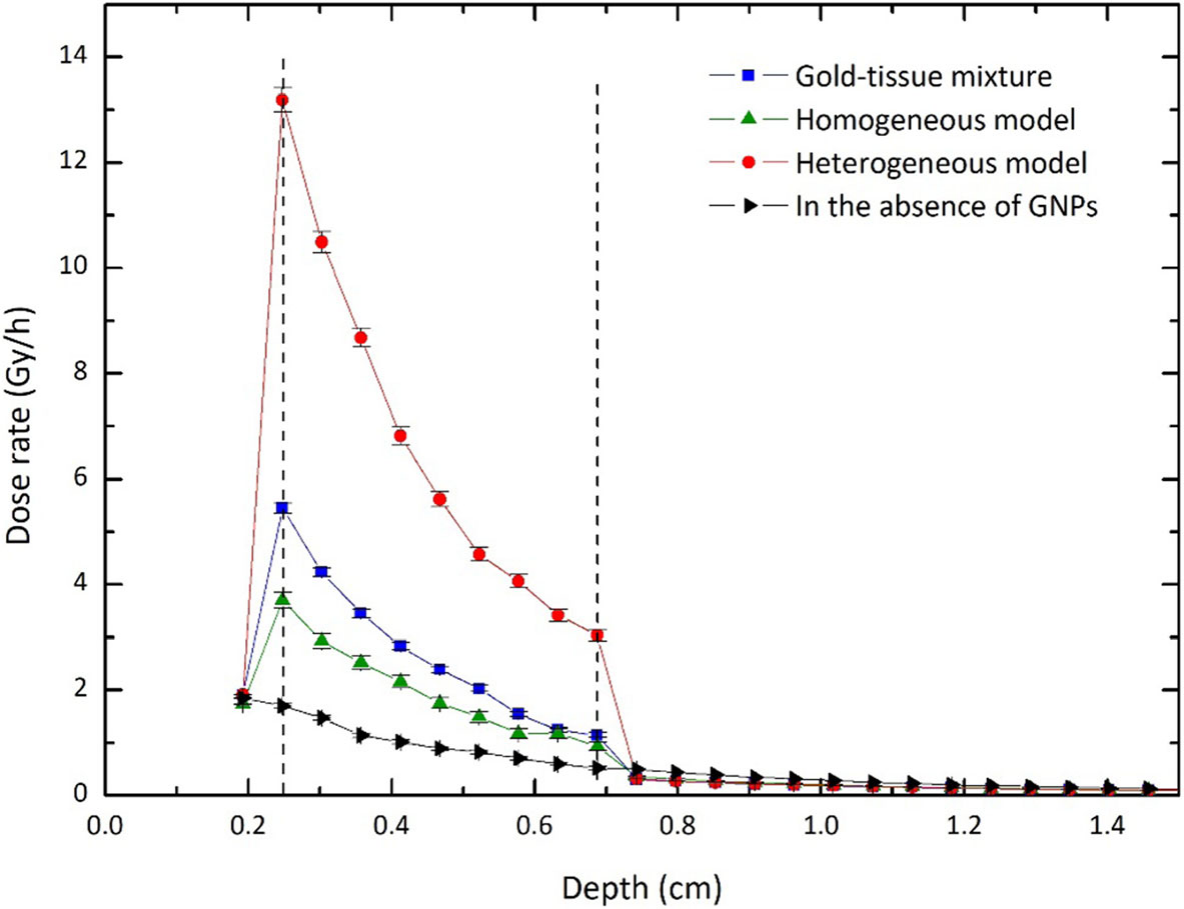
The values of the dose rate (Gy/h) calculated in the depth of human eye phantom containing the tumor filled with GNPs of three models for the concentration of 30 mg/g with 100 nm diameter. The depth-dose rates in the absence of GNPs have also been reported. Error bars indicate the relative uncertainties

Figure 3 shows the values of DEF for the tumor filled with our three models for the distribution of 100 nm GNPs with a concentration of 30 mg/g. These values have been calculated in the voxels located in the depth of the phantom (see Fig. 2a). For the same concentration and dimension of GNPs, Fig. 4 shows the depth-dose rate (in Gy/h) in the simulated phantom.

As can be seen, DEFs and dose rates corresponding to the depth of the tumor for the mixture model are larger than those of the homogeneous model. The results agree with those reported in the published works covering the overestimation of dose enhancement caused by gold-tissue mixture compared with homogeneous distribution [23, 24]. Also, DEF values for the heterogeneous model are considerably higher than those of two other models, which can be justified by the accumulation of GNPs in the volume close to the target (nucleus). In other words, in this model, the energy deposition in the nucleus due to the secondary electrons with ranges of several micrometers, generated because of the interaction of irradiated photons with GNPs, is more than two other models. For example, in the heterogeneous model, the dose rate evaluated in the first voxel of the tumor deviates from that of the sclera by about 591.8%. This deviation is about 113.4% and 185.4% for homogeneous and gold-tissue mixture models, respectively. It should be noted that the values calculated for DEF and dose rates depend on details of the models used in the problem, and our results can quantitatively change by changing the geometries and the dimensions. However, the behavior is the same.

The other interesting point in these figures is the reduction of the dose delivered to the surrounding healthy tissues in the presence of GNPs compared with the common eye plaque brachytherapy. This effect is the result of the reduction in the time that the implant needs to be used to reach the therapeutic prescribed radiation dose to the tumor (85 Gy). For example, according to Fig. 4, the apex of the tumor reaches this value in about 25 h in the heterogeneous model. This duration time is about 165 h in the absence of GNPs.

**Fig. 5:**
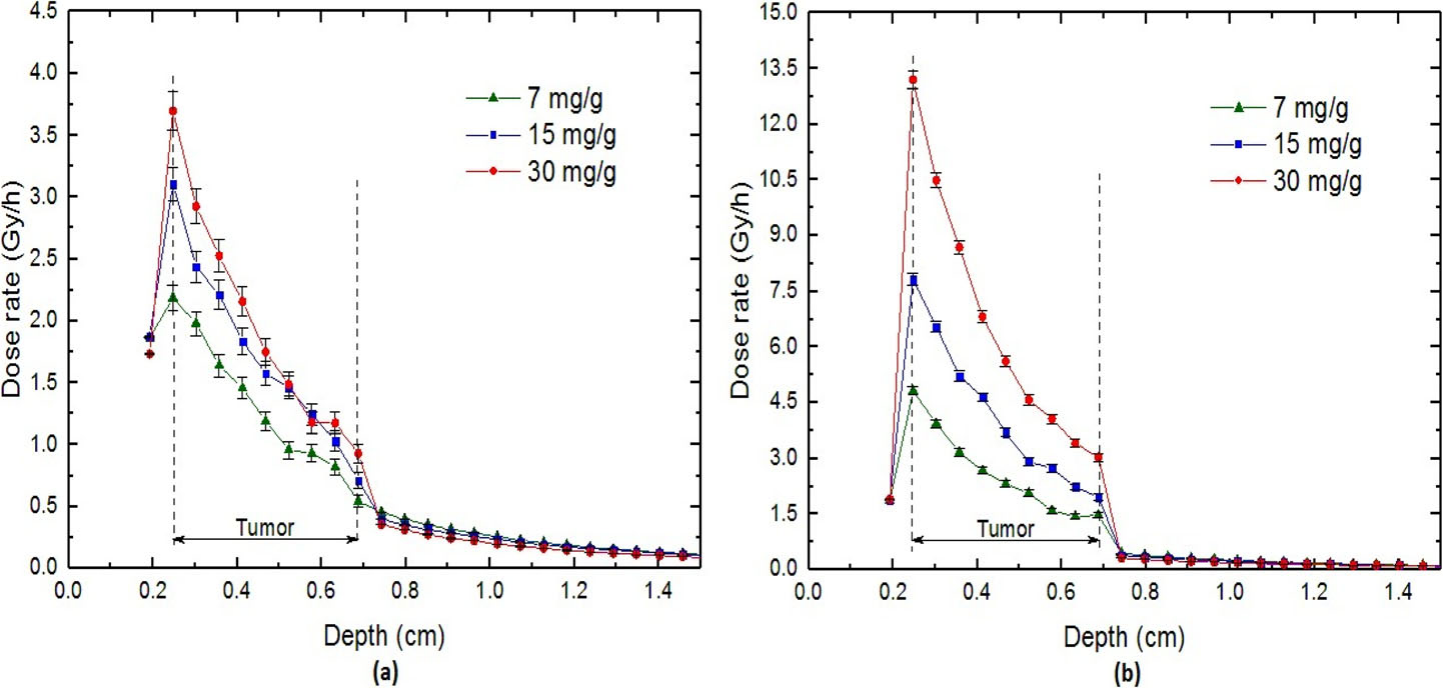
The dose rates calculated in the depth of the eye phantom containing a tumor filled with 100 nm GNPs with concentrations of 7, 15, 30 mg/g for **a** the homogeneous model. **b** The heterogeneous model

Figure 5 examines the sensitivity of the depth-dose rates to the concentration of GNPs in the tumor for homogeneous and heterogeneous distributions. In these calculations, the tested concentrations are typical values of 7, 15, and 30 mg/g, and the size of GNPs is considered to be the constant value of 100 nm in diameter. The results show that compared with the homogeneous model, the concentration is a more important factor in the dose values for the heterogeneous model. According to the results, the dose rate in the initial voxel of the tumor for the concentration of 30 mg/g deviates from that of 7 mg/g by about 174% and 69% for heterogeneous and homogeneous models, respectively. The more the concentration of GNPs increase, the more the values of dose achieves. In a constant volume, the higher density involves a larger number of GNPs. This causes increasing the interactions between photons and gold which obviously leads to growing the number of secondary electrons effective on dose enhancement. However, owing that the presence of heavy elements with a relatively high concentration into the tissue leads to cellular toxicity, increasing the concentrations to a desirable value is not biologically allowed [44–46].

To assess the effect of GNP size on the value of DEF, three diameters of 20, 50, and 100 nm have been investigated. Table 2 reports the DEFs corresponding to the homogeneous and heterogeneous models for concentrations of 7, 15, and 30 mg/g. As the results show, for a given concentration of GNPS in the homogeneous model, the values of DEF increase by increasing the size of the nanoparticle. However, this is not the only key factor for governing the dose enhancement, and the total mass of nanoparticles and the density of GNPs around the nucleus are also important. The results show that in the heterogeneous model in which the GNPs are accumulated in the small volume of cytoplasm around the target volume, the effect of GNPs’ size lessens and regardless of the size chosen for the synthesis of nanoparticles, one approximately will achieve the same dose enhancement in this model. These results, which are in agreement with those of Xie et al. [27], can be justified by increasing the number of low-energy electrons which do not contribute to the dose delivered to the nuclei because of being trapped inside the GNPs and difficulties to escape toward the target of interest.

**Table 2:** The values of DEF calculated in the nuclei of the human eye tumor for various concentrations and diameters in homogeneous and heterogeneous distributions

GNP diameter	7 mg/g		15 mg/g		30 mg/g	
	Homogeneous	Heterogeneous	Homogeneous	Heterogeneous	Homogeneous	Heterogeneous
20 nm	1.047 ± 0.005	2.580 ± 0.006	1.087 ± 0.007	4.247 ± 0.023	1.848 ± 0.011	6.770 ± 0.035
50 nm	1.206 ± 0.007	2.623 ± 0.006	1.246 ± 0.008	4.268 ± 0.023	1.583 ± 0.009	6.813 ± 0.035
100 nm	1.311 ± 0.008	2.641 ± 0.006	1.743 ± 0.010	4.257 ± 0.023	2.020 ± 0.012	6.827 ± 0.035

**Fig. 6.**
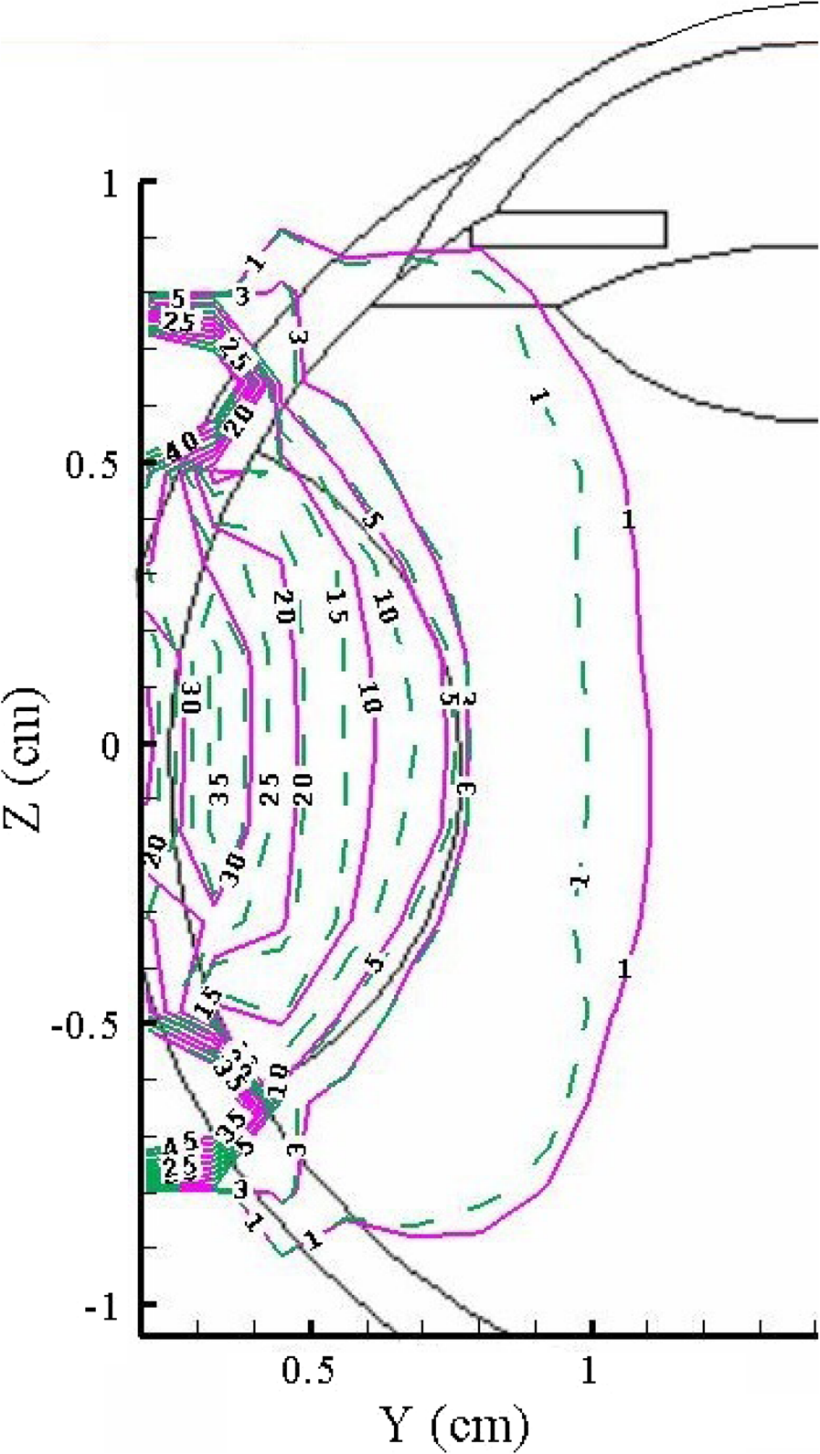
The isodose curves for the homogeneous model (solid lines) and heterogeneous model (dashed lines) in the depth of eye phantom including a tumor loaded with 100 nm GNPs of 30 mg/g

The depth-dose and depth-DEF curves reported show comparisons between the beam performances in the depth of realistic eye phantom in a specific direction for the designed models. To evaluate planar variations in absorbed dose (or DEF), the results are presented in the form of isodose curves. An example of these curves, including the results corresponding to 100 nm GNPs with a concentration of 30 mg/g, is shown in Fig. 6. As the curves show, in the case of the heterogeneous model, a given location in the tumor receives higher deposited energy compared with the same location in the homogeneous model. However, the more interesting result is that this effect will be inversed in the healthy tissue beyond the tumor, leading to the more preservation of healthy tissues. In other words, the localization of the GNPs in the small volume of cytoplasm concentrates the secondary particles in a smaller region and limits the scattering of the radiations to the non-target tissues.

**Fig. 7.**
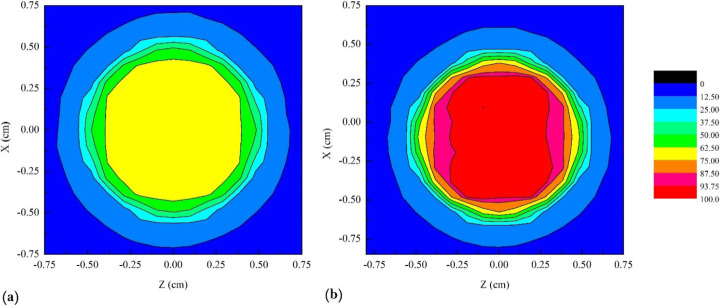
The color-filled contour plots in the X-Z plane in the central depth of the tumor (*y* = 0.45 cm) for **a** homogeneous and **b** heterogeneous models in the eye phantom including a tumor (the radius of the tumor area in the central depth is 0.5 cm) loaded with 100 nm GNPs of 30 mg/g. The color scale (in percent) has also been shown

It is also interesting to examine the dose behavior in the surface perpendicular to the axis along with the depth of the phantom (Fig. 2a). Obviously, the dose curves in the X-Z plane are depth-dependent. As an example, Fig. 7 shows the color-filled contour plots on this surface in the central depth of the tumor. As expected, the doses in both models are approximately symmetric respect to *X* and *Z* axes. Moreover, it can be found that the mean dose in the inner layers of the tumor in the heterogeneous model is larger than that of the homogeneous model, which is in agreement with the results of Fig. 5.

The main goal in radiotherapy is the achievement of high dose deposition in the tumor while sparing the surrounding healthy tissue as much as possible. To assess the performance of gold radiosensitizers in getting close to this goal, the values of DEF in various structures of the eye phantom for two distribution models are calculated. Table 3 reports these values for concentrations of 7, 15, and 30 mg/g of GNPs with a typical diameter of 50 nm.

**Table 3:** The values of DEF calculated in different structures of the eye including a tumor loaded with 50 nm GNPs with concentrations of 7, 15, and 30 mg/g for homogeneous and heterogeneous models. The last row presents the average DEFs calculated in the nuclei of the tumor

Eye structure	7 mg/g		15 mg/g		30 mg/g	
	Homogeneous	Heterogeneous	Homogeneous	Heterogeneous	Homogeneous	Heterogeneous
Vitreous	0.967 ± 0.0008	0.942 ± 0.0008	0.967 ± 0.0008	0.888 ± 0.0008	0.932 ± 0.0008	0.802 ± 0.0007
Aqueous	0.971 ± 0.004	0.952 ± 0.004	0.977 ± 0.004	0.908 ± 0.004	0.953 ± 0.004	0.832 ± 0.004
Sclera	0.996 ± 0.001	0.994 ± 0.001	0.997 ± 0.001	0.986 ± 0.001	0.994 ± 0.001	0.973 ± 0.001
Cornea	0.989 ± 0.008	0.959 ± 0.008	0.988 ± 0.008	0.917 ± 0.008	0.996 ± 0.008	0.859 ± 0.007
Iris	0.975 ± 0.011	0.968 ± 0.011	0.991 ± 0.011	0.914 ± 0.010	0.973 ± 0.010	0.834 ± 0.009
Lens	0.962 ± 0.007	0.943 ± 0.007	0.975 ± 0.007	0.887 ± 0.007	0.943 ± 0.007	0.797 ± 0.006
Tumor	1.206 ± 0.007	2.623 ± 0.015	1.246 ± 0.008	4.268 ± 0.023	1.583 ± 0.009	6.813 ± 0.035

**Fig. 8.**
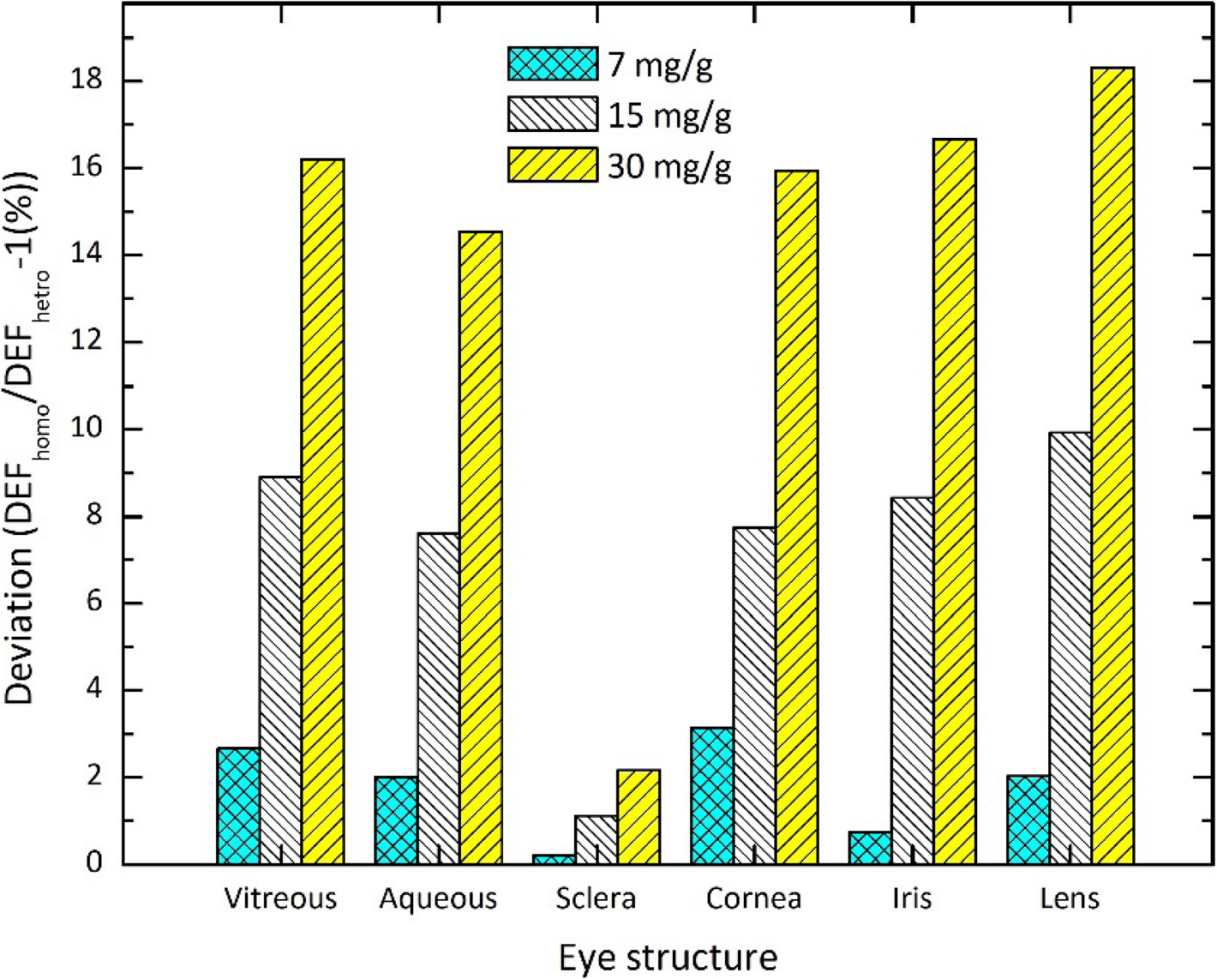
The deviation from DEF in the heterogeneous model (DEF_hetro_) of DEF in the homogeneous model (DEF_homo_) for various eye structures. These data correspond to the presence of 50 nm GNPs with concentrations of 7, 15, and 30 mg/g in the tumor

The results exhibit that the DEFs calculated in different structures of the eye containing a tumor loaded with GNPs are dependent on the distribution model. As is expected, the values of DEF in the nuclei of the tumor of the heterogeneous model are higher than those of the homogeneous model. For our tested concentrations, this difference ranges between 65.6% and 464.9%. However, the heterogeneous model leads to the decrement of the dose delivered to the sensitive structures such as lens and iris. This decrement increases with increasing the GNPs’ concentration in the tumor. For example, for 50 nm nanoparticles with the concentration of 30 mg/g, the values of DEF corresponding to the homogeneous model in the lens, iris, and vitreous deviates from those of heterogeneous model by about 18.3%, 16.6%, and 16.2%, respectively. A more detailed data on these comparisons can be found in Fig. 8, which presents the deviation from DEF in the heterogeneous model of DEF in the homogeneous model for various eye structures. These data confirm that the healthy eye structures in the homogeneous model are more at risk of receiving high doses, and consequently damage, compared with the same structures in the heterogeneous model.

## Conclusions

The importance of modeling the distribution of GNPs in the cellular level on macroscopic dose enhancement has been investigated. The results show that for a given concentration, the dose delivered to the tumor in the heterogeneous concentration is considerably larger than that of other models. For example for 100 nm nanoparticles with a concentration of 30 mg/g, the DEF in the voxel at the beginning of the tumor of the heterogeneous model deviates from those of homogeneous and gold-tissue mixture models by about 256.8% and 142%, respectively. It was found that the homogeneous model is sensitive to the size of GNPs so that the DEFs increase by increasing of the GNPs size. However, for the heterogeneous model, change in GNPs’ size will not significantly affect the energy deposition in the nucleus. Also, the results indicate that the presence of GNP within the tumor in the heterogeneous model leads to the reduction of the absorbed dose in different structures of the eye, especially in sensitive organs such as lens and iris, compared with the homogeneous model. Moreover, using GNPs reduces the time that the plaque needs to be used to reach the therapeutic prescribed radiation dose to the tumor, which decreases the radiation dose to the surrounding healthy tissues.

The results emphasize the importance of the GNPs’ distribution modeling in the cell on the overall dose enhancement of a realistic human eye phantom, as an example of macroscopic volumes extensively used for studies on gold radiosensitization. However, it is worth mentioning that while the distribution of the GNPs around the nucleus is not completely uniform, it has not been considered in our heterogeneous model. Taking into account the non-uniform distribution of GNPs in the cytoplasm offers new ways to accomplish more researches in this field.

## Data Availability

All data generated or analyzed during this study are included in this published article.
